# Endothelial cell-derived SSAO can increase MLC_20_ phosphorylation in VSMCs

**DOI:** 10.1515/biol-2021-0114

**Published:** 2021-10-21

**Authors:** Yuxing Zhang, Xiliang Zhang, Zhen Cao, Yun Huang, Yuexin Zheng, Xiaodong Yang

**Affiliations:** Department of General Surgery, The Sixth Medical Center of PLA General Hospital, Beijing 100048, People’s Republic of China

**Keywords:** SSAO, MLC_20_, iNOS, vascular hyporesponsiveness

## Abstract

Vascular hyporesponsiveness in the shock decompensation period is an important factor leading to death. Myosin light chain 20 (MLC_20_) is the main effector protein that regulates vascular reactivity. However, whether the change in semicarbazide-sensitive amine oxidase (SSAO) expression during hypoxia can change the MLC_20_ phosphorylation level, and its underlying mechanism were not clear. The amine oxidase copper containing 3 (AOC3) overexpressing adenovirus vector was constructed and transfected into rat intestinal microvascular endothelial cells (RIMECs) to overexpress SSAO, and the RIMECs were co-cultured with rat intestinal microvascular smooth muscle cells (RIMSCs). The changes in SSAO/inducible nitric oxide synthase (iNOS)/Rho associate coiled-coil containing protein kinase 1 (ROCK1) expression levels and MLC_20_ phosphorylation level were detected. Here we found that the increased SSAO by AOC3 overexpression can decrease the iNOS expression level and its activity after hypoxia. In addition, RIMSCs co-cultured with RIMECs overexpressed with AOC3 gene had significantly higher ROCK1 protein level and MLC_20_ phosphorylation level than RIMSCs co-cultured with normal RIMECs. Our study demonstrated that SSAO overexpression can significantly inhibit iNOS activity, promote RhoA/ROCK pathway activation, and increase the phosphorylation level of MLC_20_, which might be the potential mechanism in relieving the vascular hyporesponsiveness during shock decompensation.

## 1 Introduction

Post-traumatic hemorrhagic shock among trauma patients is the most important factor leading to early death in the pre-hospital setting and within 24 h of hospital admission [1]. Hemorrhagic shock after severe trauma results in deleterious clinical outcomes due to inadequate blood circulation and oxygen delivery to tissues, including multiple organ failure, coma, and possible death [2]. Many of these patients could not be treated on time and had adopted various anti-shock treatment measures such as blood transfusion, fluid replacement, and vasoactive drugs, but the death could not be avoided ultimately [3]. These patients were often in the decompensation stage of shock, or the end-stage. The most important feature of this stage is that the capillaries in the whole body are in a diastolic state that cannot be improved by vasoactive drugs, and important organs cannot get enough blood supply [4], achieving a condition called as “vascular hyporesponsiveness.”

Vascular hyporesponsiveness is a common complication of shock, which means that after severe or long-term shock, there is low vasculature response or no vascular activity [5]. This is a combination of poor tissue perfusion, refractory shock, multiple organ dysfunction syndrome, and even the primary cause of death [6]. Vascular reactivity, that is, the contraction and relaxation of vascular smooth muscle, ultimately depends on the level of phosphorylation of myosin light chain 20 (MLC_20_) [7]. Shock promotes the formation of various inflammatory factors such as tumor necrosis factor-ɑ (TNF-ɑ) [8] and changes the activity of various biological enzymes such as LDH [9], which can cause many pathophysiological changes in the internal environment. Acidosis, nitric oxide (NO), endothelin, inflammatory cytokines such as TNF-ɑ and interleukin-1β (IL-1β), endogenous opioid peptides such as β-endorphin, angiotensin (Ang), etc., are believed to reduce the phosphorylation level of MLC_20_ in different ways and cause vascular hyporesponsiveness after the shock [8].

In the initial stage of vascular smooth muscle contraction, a calcium-dependent mechanism plays a prominent role. This mechanism is completed by the norepinephrine-induced G protein (Gq)-phospholipase C-inositol triphosphate (IP3) pathway. IP3 releases Ca^2+^ from the sarcoplasmic reticulum, leading to increase in intracellular fluid Ca^2+^ concentration ([Ca^2+^] i), Ca^2+^ combined with calmodulin, activated myosin light chain kinase, phosphorylated Ser19 site on MLC_20_, increased myosin ATPase activity, phosphorylated MLC_20_, and binding of muscle actin to form a bridge that causes myofilaments to glide, and finally, the cells contract [9].

In the continuous phase of smooth muscle contraction, Ca^2+^ sensitization is considered to play a key role, which is mainly mediated by the Ras homolog family member A (RhoA)/Rho associate coiled-coil containing protein kinase (ROCK) pathway [10]. ROCK1 activated by RhoA-GTP can interact with myosin light chain phosphatase (MLCP) that binds to the myosin phosphatase target subunit 1, leading to increased phosphorylation of MLC_20_, which in turn enhances smooth muscle contraction [11].

Semicarbazide-sensitive amine oxidase (SSAO), a copper-containing amine oxidase (CuAOs) (74622 Da) consists of 763 amino acids, encoded by the amine oxidase copper containing 3 (AOC3) gene [12,13], is mainly found in the vascular endothelial cells and can be expressed and released into the blood [14,15]. Earlier, we found through a rat hemorrhagic shock model that the expression of SSAO increased during shock [16,17]. SSAO can catalyze endogenous compounds benzylamine (BZA) and methylamine (MA) and form H_2_O_2_, benzaldehyde, and formaldehyde [18]. This process can significantly reduce the expression of inducible nitric oxide synthase (iNOs) [19,20]. However, the role of the above catalytic reaction in shock is not known.

In this study, to confirm whether SSAO derived from rat intestinal microvascular endothelial cells (RIMECs) can inhibit iNOS activity and increase the level of MLC_20_ phosphorylation in rat intestinal microvascular smooth muscle cells (RIMSCs) under the hypoxic conditions, we measured SSAO, iNOS activity, and MLC_20_ phosphorylation levels by co-culturing RIMECs and RIMSCs under hypoxic conditions. In addition, the potential mechanisms in this process were investigated.

## 2 Methods

### 2.1 Animals

Twenty male SD rats were purchased from Laboratory Animal Center (SMMU, Shanghai, China).


**Ethical approval:** The research related to animal use has been complied with all the relevant national regulations and institutional policies for the care and use of animals, and were approved by the Institutional Animal Care and Use Committee of Chinese PLA General Hospital (Beijing, China), approval number SCXK(BJ)2019-0002.

### 2.2 Isolation and culture of primary cells

The isolation and culture of RIMECs and RIMSCs were performed according to previously published protocol, respectively [21].

(1) Isolation of primary RIMECs: Rats were sterilized on the abdominal wall surface with 75% of alcohol within 24 h of nascent birth, and dissected to obtain the jejunum. The jejunum was rinsed with Hank’s solution (Globo, Thermo Fisher Scientific, MA, USA), longitudinally cut, rinsed thrice, then cut into small pieces using a gun tip, and washed several times. The small pieces were then digested using collagenase (Roche, Sigma-Aldrich, MO, USA) mixture at 37°C for 1 h to expose the microvascular network, and centrifuged. The obtained pellet was cut into pieces, inoculated onto a Petri dish, and cultured at 37°C, in 5% of CO_2_.
(2) Isolation of primary RIMSCs: Within 24 h of birth, rats were disinfected on their surface with 75% of alcohol, and dissected to obtain the jejunum. This was rinsed with Hank’s solution, cut longitudinally, rinsed thrice, then cut into small pieces using the gun tip, and washed several times. The cut pieces were digested using collagenase mixture at 37°C for 2 h to expose the microvascular network, centrifuged, the pellet was then cut into pieces, and inoculated onto a Petri dish, and cultured at 37°C, in 5% of CO_2_. After the cells adhered, the cell morphology was observed, endothelial cells were scraped, and a medium (Wuhan PriCells Biomedical Technology Co., Ltd., Wuhan, China) containing specific factors was added. The third to fifth generation RIMECs were used for experimentation along with RIMSCs.

### 2.3 Packaging and extraction of adenovirus

The rat adenoviral vector pIRES-EGFP-AOC3 was constructed and packaged into virus particles to transfect the passaged RIMECs to obtain a high AOC3 gene (GeneID: 11754) expressing RIMECs. 5 × 10^5^ 293 cells were seeded in a 3.5 cm-dish and cultured at 37°C. When the confluent cells were about 70–80%, the medium was removed and replaced with fresh medium (without antibiotics) and cultured at 37°C. The plasmid was transfected with Lipofectamine 2000 and incubated overnight at 37°C. After 8 h of transfection, the cells were cultured in fresh complete DMEM medium containing 10% of FBS, 1% of antibiotics, and 1% of glutamax. After 36–48 h of transfection, the cells were digested with trypsin and cultured in 10 cm-dish. After that, fresh culture medium was changed every 2–3 days. The obvious virus spots appeared in 5–7 days, and a large number of cells disintegrated and fell off. The harvested supernatant of cell culture and cell fragments were thawed twice to lyse the cells. The virus solution was repeatedly frozen, thawed, and centrifuged at 3,000 rpm for 15 min to remove cell debris. The virus crude extract was separately packed in 1.5 mL centrifuge tube and stored at −80°C.

### 2.4 Cell culture and treatment

RIMECs were cultured in 24-well plates (Corning, NY, USA) with the density of 10^4^/cm^2^ containing DMEM (Gibco, Thermo Fisher Scientific, MA, USA) with phenol red and 20% of newborn bovine serum NBCS (Gibco, Thermo Fisher Scientific, MA, USA). After the cells were fused into a monolayer, DMEM was removed, and the cells were washed twice with Hank’s solution, followed by the addition of serum-free DMEM containing 0.1% of BSA (Sigma-Aldrich, MO, USA) without phenol red. After resting for 24 h, the medium was removed and fresh medium as before was added. This was followed by addition of Lipopolysaccharide (LPS) (10 mg/mL Sigma-Aldrich, MO, USA), and incubation in CO_2_ incubators (Thermo Scientific, Thermo Fisher Scientific, MA, USA) for 24 h. The RIMECs of experimental group were overexpressed with AOC3 transfected with adenovirus, while the control group was without transfection.

### 2.5 ELISA

The ELISA assay was performed by using the detection kit (#NBP2-80257, #NBP2-78770, Novus Biologicals, CO, USA) according to the protocol of the manufacturer. For the details, the samples were added into the wells (100 μL for each well). The plate was covered with the sealer provided in the kit and incubated for 90 min at 37°C. The liquid was removed without washing, and 100 μL of Biotinylated Detection Ab working solution was immediately added, gently mixed up, and incubated for 1 h at 37°C. It was then washed three times using the wash buffer and patted dry against clean absorbent paper. Then, 100 μL of HRP conjugate working solution was added into each well, incubated for 30 min at 37°C, and washed five times. A total of 90 μL of substrate reagent was added to each well, and incubated for about 15 min at 37°C avoiding the light. At last, 50 μL of stop solution was added to each well and the optical density (OD value) of each well was determined at once with a microplate reader set to 450 nm.

### 2.6 Western blot

The total proteins extracted from cells with RIPA lysis buffer (Beyotime, China) were quantified using a BCA Protein Assay Kit (Beyotime). A total of 50 µg of protein was separated by 10% of SDS-PAGE and transferred to a polyvinylidene fluoride (PVDF) membrane (Bio‑Rad Laboratories, Inc.). The membrane was then blocked using 5% of skim milk and incubated with SSAO (1:1,000; #PA5-104398; Gibco), iNOS (1:1,000; #PA5-37667; Gibco), ROCK1 (1:1,000; #PA5-22262; Gibco), MLC_20_ (1:1,000; #PA5-17727; Gibco), and GAPDH (1:500; #PA1-987-HRP; Gibco) antibodies overnight at 4°C. After washing three times in TBST, the membranes were incubated with anti-Rabbit IgG (H + L) HRP at a dilution of 1:3,000 in TBST containing 5% of skim milk for 2 h at 37°C. The protein bands were detected by enhanced chemiluminescence agent (Millipore, Billerica, MA, USA).

### 2.7 Establishment of co-culture model of RIMECs and RIMSCs

A double-sided co-culture model of RIMECs and RIMSCs was performed according to the previous study [21]. Transwell double-sided culture dishes with a membrane of pore size 0.4 µm (Corning, NY, USA) were used to inoculate RIMSCs at a density of 1 × 10^5^ on the reverse side of the membrane and kept in a CO_2_ incubator. After one day of incubation in the medium, the Petri dish was turned, and RIMECs (normal or overexpression treated) were inoculated on the front of the membrane at a density of 2 × 10^5^, and finally placed in a CO_2_ incubator. After three days of culture, cell fusion >90% was sufficient for use.

### 2.8 Establishment of the hypoxia cell model

The treated, double-sided Petri dish was placed in an anoxic tank, which was then filled with anoxic gases (5% of CO_2_ and 95% of N_2_), and the airway was clamped for 15 min. After 15 min, the hypoxic gases were filled again and the trachea was clamped. This was repeated five times. After the last inflation, the different times of tracheal clamping, that is, different time points of hypoxia (30, 60, 90, and 120 min) were measured.

### 2.9 SSAO activity assay

The cells were collected and then mixed in cold lysis buffer (50 mmol/L of Tris-HCl, pH 7.6, 150 mmol/L of NaCl, 5 mmol/L of CaCl_2_, 0.05% of Brij-35, 0.02% of NaN_3_, and 1% of Triton X-100) containing protease inhibitors (1 mmol/L of phenylmethylsulfonyl fluoride and 7 μg/mL of aprotinin). SSAO activity was determined radiochemically in 50 μL of cell lysis buffer, at 37°C using [^14^C]-benzylamine (3 mCi/mmol; Amersham) as substrate. Protein was quantified using the BCA method (Pierce, Rockford, Ill).

### 2.10 Quantitative RT-PCR

Total RNA was extracted from cells using TRIzol reagent (Invitrogen; Thermo Fisher Scientific Inc.). RNA was reverse‐transcribed to complementary DNA using a PrimeScript RT Reagent Kit (Takara Biotechnology Co., Ltd., Dalian, China). Quantitative RT‐PCR was performed using SYBR Premix Real‐time PCR Reagent (Takara Biotechnology) on an ABI 7500HT instrument (Bio‐Rad, Hercules, CA). For mRNA detection, the primers used in this study were as follows: AOC3 (forward: 5′-TACAACCACTGCACTACCTG-3′, reverse: 5′-TGGAATGCTTGAAGGCTGCT-3′) and GAPDH as an internal control (forward: 5′-ACCTGACCTGCCGTCTAGAA-3′, reverse: 5′-TCCACCACCCTGTTGCTGTA-3′). Each experiment was performed in triplicate, and the data were analyzed by the 2^−△△Ct^ method.

### 2.11 Statistical analyses

SPSS 16.0 software was used for statistical analyses, and the data were represented as mean value ± standard deviation. Two independent sample *t*-tests were performed to compare the two groups, and one-way ANOVA was used for comparison between multiple groups. *P* < 0.05 was considered statistically significant.

## 3 Results

### 3.1 Construction and detection of adenovirus vectors containing AOC3

**Figure 1:**
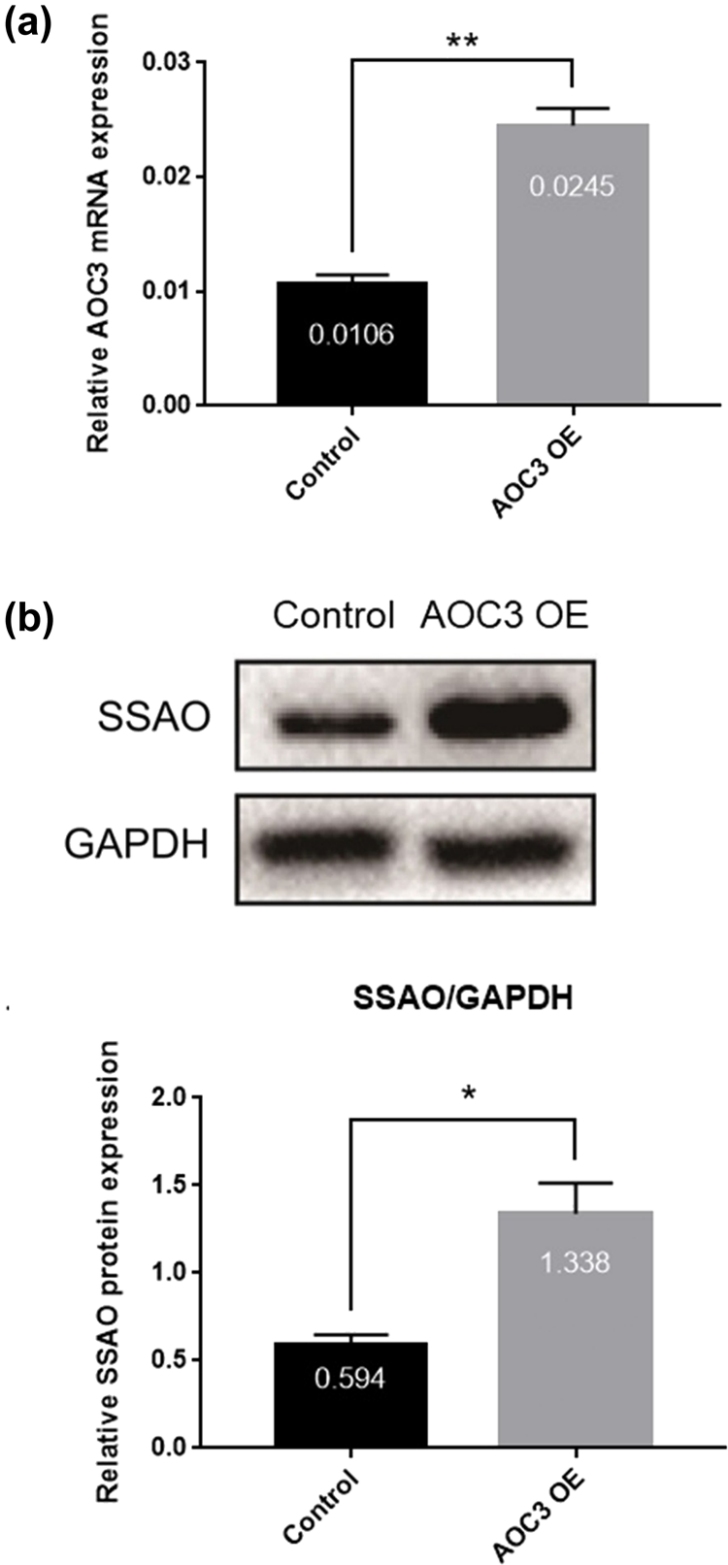
Detection of AOC3 overexpression in RIMECs by adenovirus. (a) AOC3 mRNA expression in RIMECs as determined by qRT-PCR. GAPDH was used as an internal control. (b) SSAO protein expression in RIMECs was determined by Western-Blot. GAPDH was used as an internal control (*P < 0.05, **P < 0.01, and ***P < 0.001).

**Figure A1:**
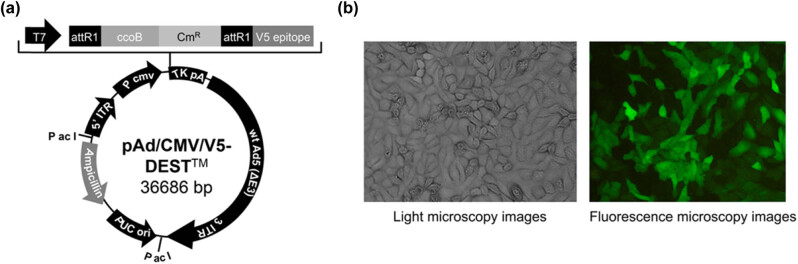
Construction of adenovirus vector for AOC3 overexpression. (a) Adenoviral vector (pAD/CMV/V5-DEST) for AOC3 overexpression. (b) Light and fluorescence microscopic images confirmed the successful transfection of RIMECs with pAd-AOC3-IRES-EGFP (magnification, 200×).

To investigate the role of SSAO in hypoxia, the pAd-AOC3-IRES-EGFP adenoviral vector was successfully constructed (Figure A1a) and the RIMECs were successfully transfected with high efficiency by using the adeno-associated virus (Figure A1b). The qRT-PCR and western blotting assays showed that RIMECs transfected with the pIRES-EGFP-AOC3 adenovirus vector had significantly higher expression of AOC3 mRNA (Figure 1a) and SSAO protein (Figure 1b).

### 3.2 SSAO can regulate LPS-induced iNOS expression in RIMECs

**Figure 2:**
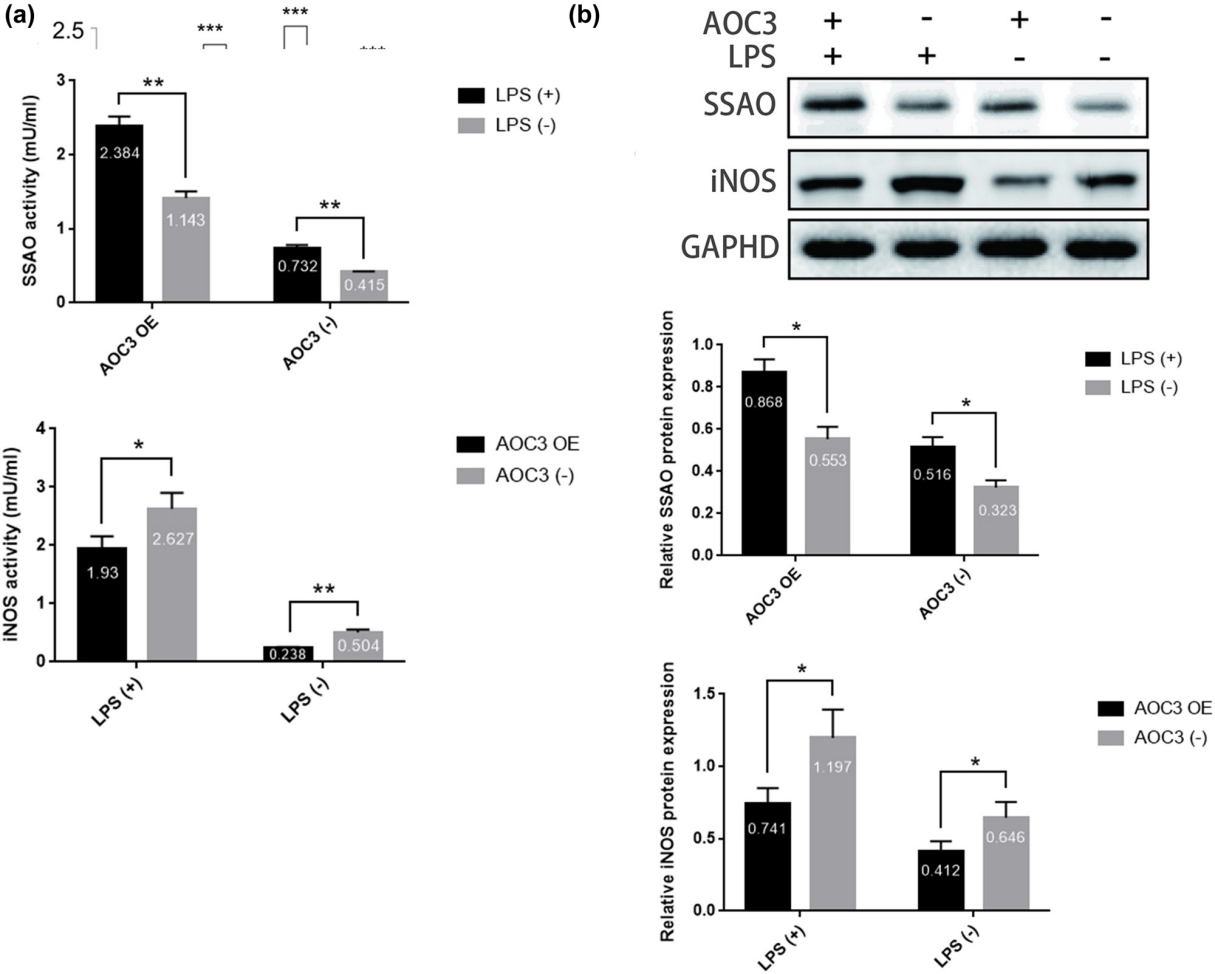
SSAO can regulate LPS-induced iNOS expression in RIMECs. (a) SSAO and iNOS activity in RIMECs culture medium were determined using Fluoro SSAO™ Fluorescent SSAO Detection kit and PathScan® Total iNOS Sandwich ELISA Kit. (b) SSAO and iNOS protein expression in RIMECs, as determined by the western blotting. GAPDH was used as an internal control (*P < 0.05, **P < 0.01, and ***P < 0.001).

To explore the potential role of SSAO in regulating iNOS expression level and its activity during the process of hypovascular responsiveness, the inflammatory factor LPS was used to induce the high expression of iNOS in vascular smooth muscle cells. In this study, the administration of inflammatory factor LPS can lead to significantly higher expression of iNOS in RIMECs than that without LPS, accompanied with significantly enhanced iNOS activity. Concurrently, the expression level and activity of SSAO in control and AOC3-overexpressed RIMECs with LPS were significantly higher than those without LPS (Figure 2a). In addition, the expression level of iNOs and its enzyme activity in RIMECs with AOC3 gene overexpression were significantly reduced compared to those with control RIMECs (Figure 2b).

### 3.3 Overexpression of AOC3 regulates iNOS expression and its activity in RIMECs under hypoxia

**Figure 3:**
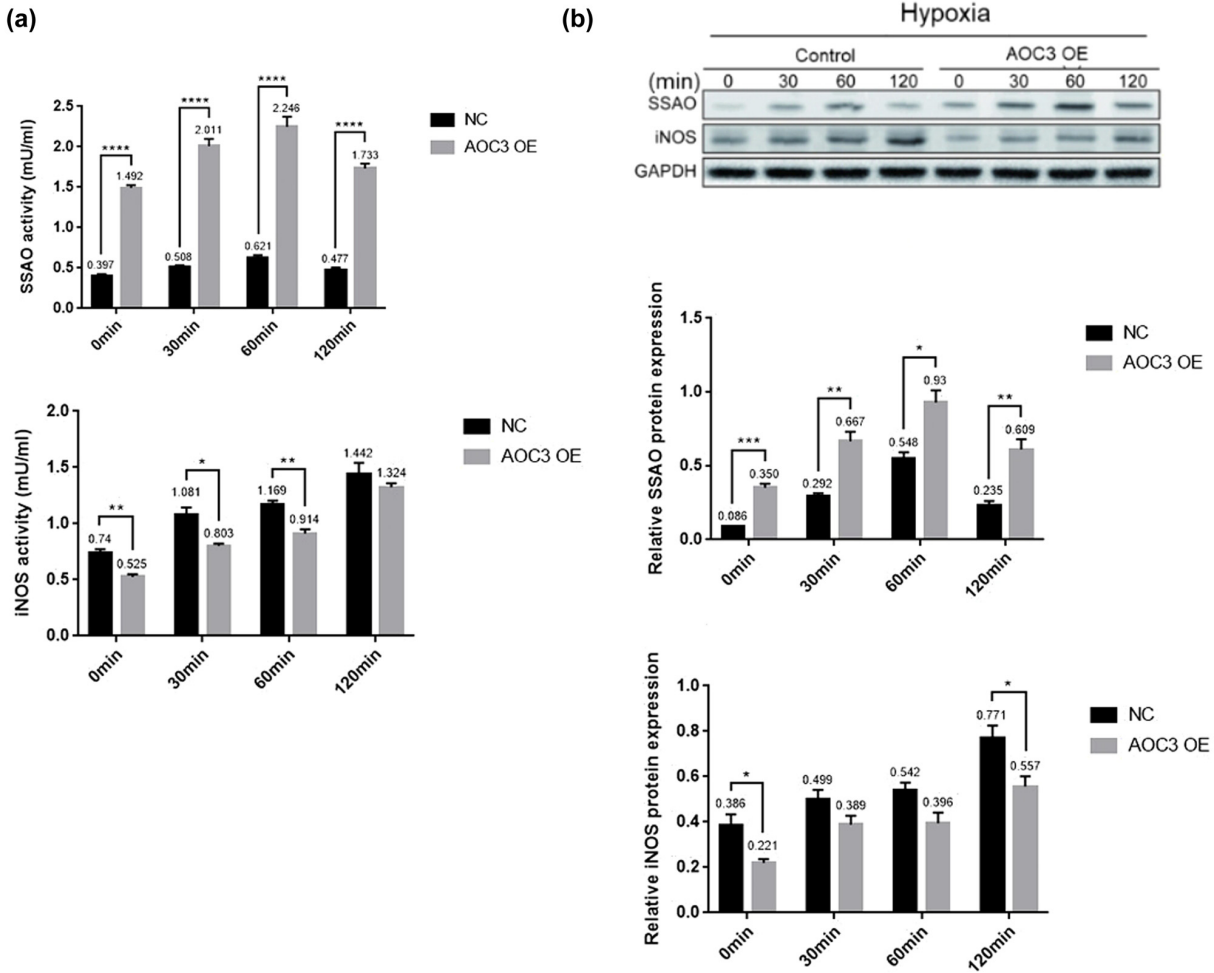
Overexpression of AOC3 regulates iNOS expression and its activity in RIMECs under hypoxia. (a) SSAO and iNOS activity in RIMECs and RIMSCs co-culture medium were determined using Fluoro SSAO™ Fluorescent SSAO Detection kit and PathScan® Total iNOS Sandwich ELISA Kit. (b) SSAO and iNOS protein expression in RIMECs, as determined by the western blotting. GAPDH was used as an internal control (*P < 0.05, **P < 0.01, and ***P < 0.001).

Our previous experiments confirmed that the expression of SSAO in vascular endothelial cells of rats is gradually increased under the condition of hemorrhagic shock [16,17]. To further study the role of SSAO in regulating iNOS expression level under hypoxia, here the SSAO expression level and its activity in RIMECs were detected and we found that both of them were gradually increased at 30 and 60 min after hypoxia, but decreased at 120 min, with a peak at 60 min (Figure 3a), while the expression level of iNOS protein and its enzyme activity showed only a gradual increasing trend (Figure 3b). Under hypoxic conditions, the SSAO expression level and its activity in RIMECs overexpressed with AOC3 gene were observed at various time points and were found significantly higher than those in the control RIMECs, while the iNOS expression and its activity were significantly lower than those in control RIMECs at different time points (Figure 3b).

### 3.4 Overexpression of AOC3 increases the ROCK1 expression and phosphorylation of MLC_20_ in RIMSCs under hypoxia

**Figure 4:**
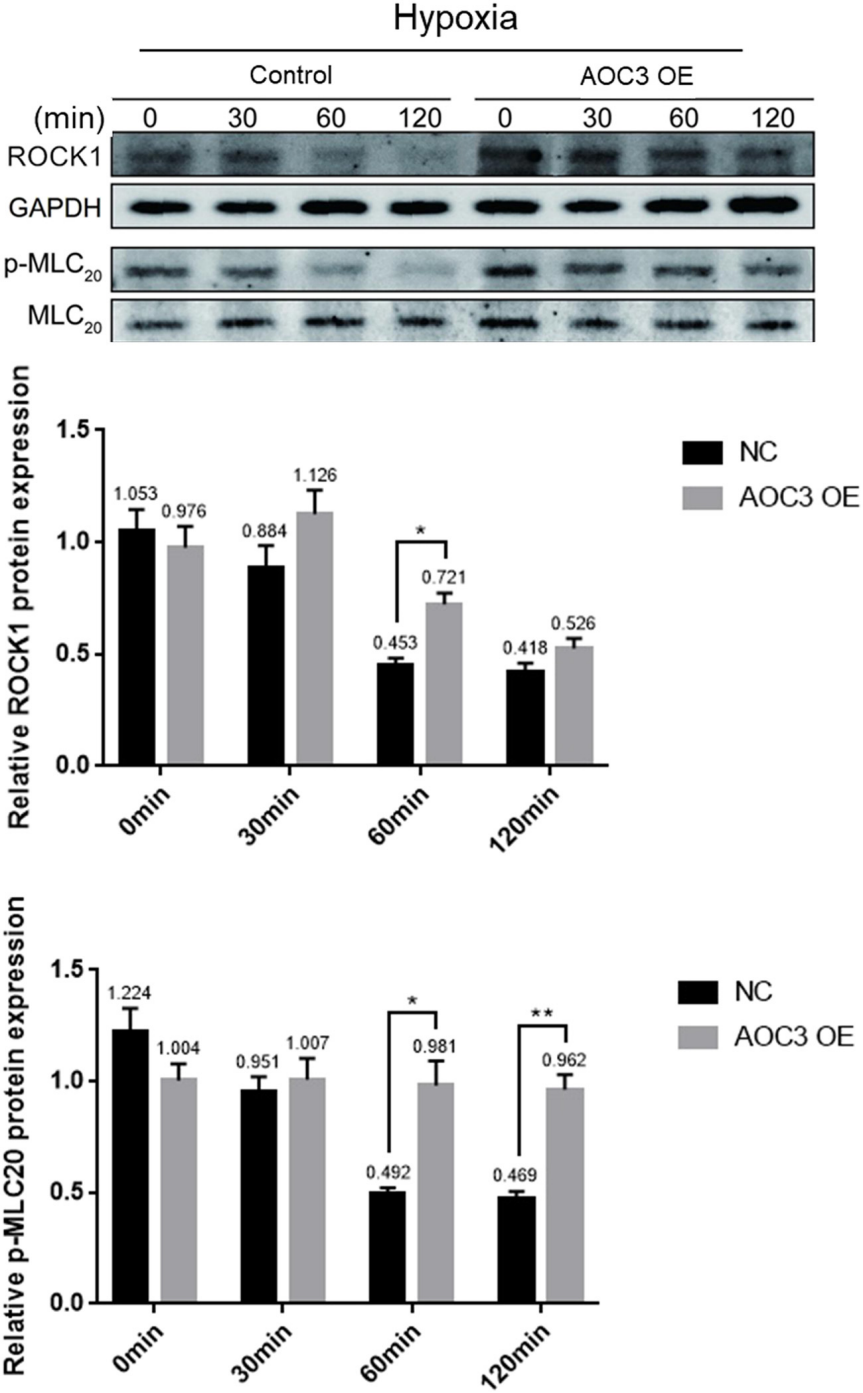
Overexpression of AOC3 increases the ROCK1 expression and phosphorylation of MLC_20_ in RIMSCs under hypoxia. ROCK1 and p-MLC_20_ protein expression in RIMSCs were determined by western blotting. GAPDH and MLC_20_ were used as internal controls, respectively (*P < 0.05, **P < 0.01, and ***P < 0.001).

RhoA/ROCK signaling pathway plays an important role in the continuous phase of Ca^2+^-dependent smooth muscle contraction. In order to confirm whether SSAO from vascular endothelial cells can increase the level of MLC_20_ phosphorylation under hypoxia, and whether it is mediated by RhoA/ROCK pathway, we detected the expression level of ROCK1 and the phosphorylation level of MLC_20_ in RIMSCs co-cultured with RIMECs under hypoxia. After 30 min of hypoxia, RIMSCs co-cultured with RIMECs overexpressed with AOC3 gene had significantly higher ROCK1 protein levels than RIMSCs co-cultured with control RIMECs, and after 60 min of hypoxia, RIMSCs co-cultured with RIMECs overexpressed with AOC3 gene showed higher level of MLC_20_ phosphorylation than that observed in RIMSCs co-cultured with control RIMECs (Figure 4).

## 4 Discussion

In this study, we found the increased SSAO and its activity in RIMECs after hypoxia. Upregulation of the SSAO level by AOC3 overexpression in RIMECs significantly decreased the expression level and enzyme activity of iNOS compared to control. In addition, RIMSCs co-cultured with RIMECs overexpressed with AOC3 gene had significantly higher ROCK1 protein level and MLC_20_ phosphorylation level than RIMSCs co-cultured with normal RIMECs. The SSAO enzyme is mainly expressed by RIMECs and can be released in the blood to play its catalytic role [14,22]. Previous research demonstrated that the metabolites of BZA or MA catalyzed by SSAO could also significantly decrease LPS-induced iNOS and cyclo-oxygenase-2 (COX-2) expression, as well as NO production, which might be useful for normalization of glucose disposal during endotoxemia [20], while its role in hemorrhagic shock are largely unknown. Here we co-examined RIMECs with RIMSCs by co-culturing and found that the increased expression level of SSAO in RIMECs can restrain the LPS and hypoxia-induced iNOS expression and might modulate the reactivity of RIMSCs *in vitro*. However, another study reported that the reduced AOC3 mRNA and vascular adhersion protein (VAP‑1) levels in hepatic and intestinal tissues from rats following hemorrhagic shock appeared to improve survival in animals not receiving resuscitation following hemorrhagic shock [17]. The inconsistency between *in vitro* and *in vivo* results need to be further verified by future experiments.

Although both the iNOS and MLC_20_ were reported showing the association with vascular hyporesponsiveness [23,24], the relationship between iNOS and MLC_20_ in the hemorrhagic shock was not reported. Here we found that both p-MLC_20_ and ROCK1 – that promoted phosphorylation of MLC_20_ – showed a significant decline, indicating that iNOS can inhibit RhoA activation and MLC_20_ phosphorylation under hypoxic conditions, while a large amount of SSAO expression significantly inhibited this effect. Our study also confirmed that when RIMECs overexpress SSAO, iNOS, which was originally expressed in large quantities due to the addition of inflammatory factors such as LPS, exhibited significantly reduced expression and enzyme activity. Thus, we conclude that SSAO can delay the RIMSCs RhoA activation and causes a decline in MLC_20_ phosphorylation levels in hypoxia by inhibiting iNOS activity expressed by RIMECs, thereby alleviating the hyporesponsiveness state.

The inhibition of iNOS expression by SSAO may be related to the catalytic products of SSAO. The endogenous substrates of SSAO, BZA, and MA are catalyzed by SSAO to form H_2_O_2_, benzaldehyde, and formaldehyde, and this process can significantly reduce the expression of iNOS, COX-2, and other cytokines induced by LPS [20]. Besides directly inhibiting iNOS expression, as an important active component of reactive oxygen species, H_2_O_2_ may also improve vascular reactivity during shock by (i) inducing muscles by stimulating IP3 and ryanodine receptors [Ca^2+^] i released from plasma network [25,26]], (ii) acting on mitogen-activated protein kinase, tyrosine kinase, ROCK1, and transcription factor hypoxia inducible factor-1, causing [Ca^2+^] i and increased calcium sensitivity [27], and (iii) direct activation of the RhoA-ROCK pathway by activating Src-family kinase-Rho guanine nucleotide exchange factor 1 [28]. These studies indicate that SSAO and its catalytic products may alleviate the decrease in vascular reactivity during shock decompensation by inhibiting iNOS, promoting [Ca^2+^] i release, increasing calcium sensitivity, and directly activating the RhoA-ROCK pathway. But which mechanism plays a major role needs to be further studied.

## 5 Conclusion

In summary, we confirmed that the increased SSAO expression in RIMECs under the hypoxic condition can inhibit iNOS expression and its activity through regulating the RhoA-ROCK pathway and increasing MLC_20_ phosphorylation level, which might alleviate the hyporesponsiveness of blood vessels in the decompensated stage of shock.
